# Is primary chemotherapy effective in large hydatid cyst of liver?

**DOI:** 10.4103/0971-9261.54806

**Published:** 2009

**Authors:** A. N. Gangopadhyay, Punit Srivastava, Vijai D. Upadhyaya, Zaheer Hasan

**Affiliations:** Department of Pediatric Surgery, IMS, BHU, Varanasi, India

Sir,

Surgery remains the treatment of choice for hydatid cyst of the liver. Percutaneous drainage and medical treatment using benzimidazole compounds (albendazole, mebendazole) are the other options. We herein report a patient with hydatid cyst of the liver treated nonoperatively with albendazole because he was reluctant to surgery. The patient improved dramatically and was asymptomatic after 4 months of therapy.

A 7-year-old boy presented with pain in right upper abdomen of 3 months duration. There was no history of fever, jaundice and trauma. The abdominal examination revealed mild hepatomegaly. The hematological and biochemical investigations were within normal limits. An ultrasonography was suggestive of hydatid cyst with a size of 11.5 × 9.5 cm in the left lobe of liver. The diagnosis was confirmed with plasma IgG anti-echinococcal antibody titers, which was 15.27U/mL at the time of presentation. The patient was planned for elective operation with pre-operative chemotherapy (albendazole) for 3 weeks. The patient was subjected to repeat ultrasound, which revealed that the size of cyst reduced to almost half measuring 6.5 × 3.9 cm. At this stage, the patient was reluctant for surgical intervention because he was feeling better, asymptomatic and also because of financial problems. We decided to continue the medical treatment under strict follow up. Repeat ultrasonography revealed a completely shrunken cyst. Repeat plasma IgG anti-echinococcal antibody titer is now in normal range. The patient took albendazole for 4 months and was asymptomatic at 6-month follow up.

In contemporary practice, the indications of benzimidazole therapy are inoperable primary hepatic hydatidosis, multiple cysts in two or more organs, multiple small liver cysts, cysts located in deep hepatic parenchyma, prevention and management of secondary hydatidosis, management of recurrent hydatidosis, unilocular cysts in unfit elderly patients, in adjunct therapy with surgery or percutaneous interventions, pulmonary echinococcosis, and long-term treatment for cystic echinococcosis in specific organs such as the bone, brain, or eye. The usual dosage of albendazole is generally suggested to be 10-15 mg kg per day[1] in two equal doses in courses of 3 months, separated by intervals of 1 or 2 weeks.[2,3] Horton[4] classified the clinical outcome of patients treated with albendazole as cure, improvement, no change and worsening and reported that 30% of the patients were cured, 30%-50% had improvement and 20%-40% had no change. Gil-Grande *et al*,[5] suggested that initial medical therapy to be a good alternative to surgical therapy in uncomplicated hepatic hydatid cysts in their study of 55 patients. Keshmiri *et al*.[6] treated 29 patients with 240 cysts intermittently for 6 months and reported a cure rate of 10%, a reduction in cyst size of 60%, and an improvement in morphological appearance by 62%. Senyüz *et al*.[7] concluded in their study that medical treatment of the hydatid disease must not be limited to only small-sized cysts. Before proceeding to surgery, many hydatid cysts, even when huge in size, may respond to the treatment either with shrinkage of the germinative membranes or a deposition of calcium in their walls although such shrinkage of the cyst may take 1 to 2 years. Therefore, any unnecessary risk of surgical intervention can be eliminated. Based on these studies, we have tried the medical treatment for single large uncomplicated hydatid cyst because the patient was reluctant to surgical intervention. The patient was completely asymptomatic after 4 months of albendazole therapy. Therefore, we suggest that medical therapy is a good cost-effective alternative for surgical intervention, especially when patient is reluctant to surgery or he is not fit for surgery.
